# Transcriptomic responses of Mediterranean sponges upon encounter with symbiont microbial consortia

**DOI:** 10.1186/s12864-024-10548-z

**Published:** 2024-07-07

**Authors:** Angela Maria Marulanda-Gomez, Marta Ribes, Sören Franzenburg, Ute Hentschel, Lucia Pita

**Affiliations:** 1 RD3 Marine Ecology, RU Marine Symbioses, GEOMAR Helmholtz Centre for Ocean Research Kiel, Kiel, Germany; 2 Institut de Ciències del Mar, ICM – CSIC, Barcelona, Spain; 3 Research Group Genetics and Bioinformatics/Systems Immunology, Institute of Clinical Molecular Biology, Christian-Albrechts-Universität Kiel, Kiel, Germany; 4 Christian-Albrechts-Universität Kiel, Kiel, Germany

**Keywords:** Animal-microbe interactions, Microbial consortia, HMA-LMA sponges, Immune receptors, NLRs, RNA-Seq, Differential gene expression, Symbiosis

## Abstract

**Background:**

Sponges (phylum Porifera) constantly interact with microbes. They graze on microbes from the water column by filter-feeding and they harbor symbiotic partners within their bodies. In experimental setups, sponges take up symbionts at lower rates compared with seawater microbes. This suggests that sponges have the capacity to differentiate between microbes and preferentially graze in non-symbiotic microbes, although the underlying mechanisms of discrimination are still poorly understood. Genomic studies showed that, compared to other animal groups, sponges present an extended repertoire of immune receptors, in particular NLRs, SRCRs, and GPCRs, and a handful of experiments showed that sponges regulate the expression of these receptors upon encounter with microbial elicitors. We hypothesize that sponges may rely on differential expression of their diverse repertoire of poriferan immune receptors to sense different microbial consortia while filter-feeding. To test this, we characterized the transcriptomic response of two sponge species, *Aplysina aerophoba* and *Dysidea avara*, upon incubation with microbial consortia extracted from *A. aerophoba* in comparison with incubation with seawater microbes. The sponges were sampled after 1 h, 3 h, and 5 h for RNA-Seq differential gene expression analysis.

**Results:**

*D. avara* incubated with *A. aerophoba*-symbionts regulated the expression of genes related to immunity, ubiquitination, and signaling. Within the set of differentially-expressed immune genes we identified different families of Nucleotide Oligomerization Domain (NOD)-Like Receptors (NLRs). These results represent the first experimental evidence that different types of NLRs are involved in microbial discrimination in a sponge. In contrast, the transcriptomic response of *A. aerophoba* to its own symbionts involved comparatively fewer genes and lacked genes encoding for immune receptors.

**Conclusion:**

Our work suggests that: (i) the transcriptomic response of sponges upon microbial exposure may imply “fine-tuning” of baseline gene expression as a result of their interaction with microbes, (ii) the differential response of sponges to microbial encounters varied between the species, probably due to species-specific characteristics or related to host’s traits, and (iii) immune receptors belonging to different families of NLR-like genes played a role in the differential response to microbes, whether symbionts or food bacteria. The regulation of these receptors in sponges provides further evidence of the potential role of NLRs in invertebrate host-microbe interactions. The study of sponge responses to microbes exemplifies how investigating different animal groups broadens our knowledge of the evolution of immune specificity and symbiosis.

**Supplementary Information:**

The online version contains supplementary material available at 10.1186/s12864-024-10548-z.

## Background

Over the last decades, animals were recognized as “metaorganisms” or “holobionts” which encompass the multicellular host and its microbial symbionts, such as bacteria, archaea, viruses, protists, fungi, and algae, [1–4]. Symbionts participate in the general fitness of the host by contributing to developmental cues, nutrient provision, potential metabolic expansion, reproduction, and defensive traits [5–8]. Microbes thus provide adaptive advantages and shape animal evolution [9, 10]. These close and complex host-microbe interactions require fine-tuned communication between partners which is now known to be orchestrated by the host immune system [11–14].

How does immunity differentiate pathogens to eliminate from symbionts to acquire/maintain, and how does it safeguard homeostasis within the host? This question is a frontier in symbiosis research. Evidence suggests that animals first sense microbe-associated molecular patterns (MAMPs), such as lipopolysaccharide, peptidoglycan, or flagellin, via pattern recognition receptors (PRRs) [15]. However, from that ligand-receptor encounter, a pathogenic microbe elicits inflammatory responses eliminating the intruder, whereas a symbiont promotes tolerance and colonization [16, 17]. In aquatic invertebrates, which are in constant contact with microbes in the water, this specific microbial recognition must be possible, even if they lack an adaptive immune system. Their large and strikingly complex repertoires of PRRs [18–22] potentially play a role in microbial discrimination [21, 23–25]. For example, the coral *Montipora aequituberculata* responds to potentially pathogenic and commensal bacteria (*Vibrio coralliilyticus* and *Oceanospirillales* sp., respectively) by regulating the expression of Toll-like receptors and via differential upregulation of G protein–coupled receptors [26]. On the other hand, the freshwater snail *Biomphalaria glabrata* recognizes different pathogens by different sets of PRRs belonging to the calcium-dependent lectin family and via enzymes and non-canonical immune components, like extracellular actin [27]. However, the mechanisms of microbial discrimination and specificity in aquatic invertebrates, particularly in the context of symbiosis, are still not well understood and remain to be elucidated.

As arguably the earliest branching metazoans [28, 29], sponges (phylum Porifera) offer the opportunity to study the evolution of innate immune specificity and mechanisms of animal-microbe interactions. As active filter-feeders pumping thousands of liters of seawater per day through their aquiferous system, sponges constantly encounter microbes from the seawater, but, at the same time, harbor specific and complex microbial communities occurring mainly extracellularly, within the sponge mesohyl matrix [30, 31]. Early experimental evidence showed that sponges preferentially take up seawater microbial consortia (i.e., bacterioplankton) over their own sponge symbiont consortia [32–34]. In these incubation experiments, seawater bacteria concentration in water decreased over time, the expected trend if sponges are actively filter-feeding. And indeed, labelled seawater bacteria were detected inside sponge cells. However, symbiont concentration in the water remained stable over time and none or few labelled symbiotic bacteria were found inside the sponge, suggesting they were avoiding being taken up or phagocytized by the sponge [32–34]. These studies have served as evidence that the animal can differentiate between different microorganisms. The high diversification of PRRs (e.g., NLRs: Nucleotide Oligomerization Domain (NOD)-Like Receptors and SRCRs: Scavenger Receptor Cysteine-Rich) in sponge host draft genome and transcriptome assemblies [35–38] suggest their potential to recognize different and specific microbial ligands [39]. In fact, there is initial experimental evidence in sponges regulating the expression of receptors, in particular NLRs, SRCRs, and G-protein coupled receptors (GPCRs), as well as of genes related to apoptosis and phagocytosis upon encounter with microbial elicitors [40–43].

Our study aims to better understand the underlying molecular mechanisms of bacterial discrimination in sponges. We characterized the host response of *D. avara* and *A. aerophoba* upon encounter to seawater- and sponge-derived microbial consortia by RNA-Seq differential gene expression analysis. These two species were chosen based on the previous comparative study of microbial discrimination by Wehrl et al. [32, 33], which showed that *A. aerophoba* preferentially retains seawater bacteria (food) over its own symbionts and that the symbiont consortia extracted from *A. aerophoba* was taken up and incorporated in a lesser proportion in *A. aerophoba* than in *Dysidea avara*. These two species are representatives of high microbial and low microbial abundance (HMA and LMA) sponges. HMA sponges, such as *A. aerophoba*, contain more diverse and abundant microbial communities than LMA species, such as *D. avara* [44–47]. This HMA-LMA status is a long-recognized dichotomy in sponge-microbe symbiosis reflecting particular signatures in the structure and persistence of the symbiosis as well as physiological differences such as density of the mesohyl and pumping rates [48–51]. We incubated *D. avara* (LMA) and *A. aerophoba* (HMA) individuals with either sponge-associated symbiotic consortia or with microbial consortia enriched from natural seawater following a similar experimental approach as in Wehrl [33]. Sponge symbionts remain uncultured; thus, the symbiont consortium was obtained from *A. aerophoba* by differential centrifugation, a physical separation used to enrich symbiotic fractions [32, 52]. We collected samples at 1 h, 3 h, and 5 h from the start of the incubation. We hypothesized that sponges may rely on differential expression of immune receptors for microbial discrimination, as poriferan receptors are highly diverse [35–38] and observed to be regulated upon encounter with microbial elicitors [42, 43]. This is based on previous experiments showing that sponges challenged with bacterial elicitors respond by regulating the expression of immune receptors belonging to the families of NLRs, SRCRs, and GPCRs [42, 43]. Furthermore, we expected differentially expressed genes to show lower expression levels upon symbiotic than seawater microbial consortia treatment based on the assumption that symbiotic microbes silence components of the immune system to avoid phagocytosis [53]. Finally, we hypothesized that *D. avara* recognizes the symbiont consortium as “foreign/unknown”, as this consortium was extracted from *A. aerophoba*. Hence, a stronger response (i.e., higher number of differentially expressed genes) was expected in *D. avara* than in *A. aerophoba* upon encounter with *A. aerophoba*-symbiont consortium compared to seawater microbial consortium.

## Materials and methods

### Sponge collection

Specimens of the Mediterranean sponge species *Aplysina aerophoba* (Nardo, 1833) and *Dysidea avara* (Schmidt, 1862) were collected via SCUBA diving at the coast of Girona (Spain) in March 2015 (42.29408 N, 3.28944 E and 42.1145863 N, 3.168486 E; respectively). A total of 10 individuals were collected per species. Sponges were then transported to the Experimental Aquaria Zone (ZAE) located at the Institute of Marine Science (ICM-CSIC) in Barcelona (Spain) and were placed in separated 6 L aquaria in a flow-through system with direct intake of seawater. Temperature and light conditions were set up mimicking natural conditions. Sponges were maintained under these conditions during 10–12 days for acclimation.

### Experimental setup

The experiment was conducted consecutively for each sponge species (end of March for *A. aerophoba*, beginning of April for *D. avara*). Before the microbial exposure experiments, sponges were kept overnight in 1 μm-filtered seawater and an additional 0.1 μm-filter was applied for 3 h before the experiments with the aim to reduce microbial load in seawater to a minimum. The flow-through was stopped during the experiment, but small aquarium pumps (Eheim) ensured the mixing of the water in the aquarium. Sponges were incubated with either microbial seawater consortia or symbiont consortia that had been prepared following the protocols below. The concentration of microbes in the tanks before adding each treatment, as well as the concentration of the microbial consortia stocks were estimated via flow cytometry (see details in supplementary information, Text S1 and Fig. S1). Before starting the experiment, the concentration of microbes was approx. 10^3–4^ bacteria mL^− 1^ and the microbial consortia stocks were adjusted to reach 10^5–6^ bacteria mL^− 1^ final concentration in the experimental tanks (Fig. S1). This final concentration represents typical microbial densities in seawater in the area where the sponges were collected [54, 55]. Sponge specimens that were actively pumping, as visually assessed by the presence of an open oscula, were randomly assigned to each treatment (*n* = 5 individuals per treatment). For each individual, tissue samples were collected at 1 h, 3 h, and 5 h after adding the microbial consortia to the experimental tanks. Then, the tissue was placed in RNAlater at 4 °C overnight and stored at -80 °C until processing.

### Symbiont consortia preparation

The *A. aerophoba*-symbiont fraction was obtained as described in Wehrl et al. [32]. Briefly, 20 g of sponge tissue was collected from living individuals that were sampled at the same time and in the same site where the sponge collection was performed. The tissue was cleaned off debris, rinsed in sterile, ice-cold Ca- and Mg-free artificial seawater (CMFASW) with EDTA (as in [56]), incubated for 30 min at 4 °C, and then homogenized with a mortar and pestle. After filtration through 100 μm-Nitex, the suspension was centrifuged twice at 4 °C, 400 g for 20 min to remove sponge cells, which remained in the pellet. The supernatants were combined and centrifuged at 4 °C, 4000 g for 20 min to obtain a bacterial pellet. This pellet was washed twice in ice-cold CMFASW and recovered again by centrifugation. Finally, the bacterial pellet was resuspended in sterile ice-cold CMFASW. Symbiont extraction from *D. avara* was not possible because this species represents an LMA sponge and we could not obtain enough microbial extracts for the incubations.

### Seawater microbial consortia preparation

Seawater microbial consortia were enriched from seawater from the aquaria setup (a flow-through system with direct intake of natural seawater), following the protocol by Wehrl et al. [32]. In short, Marine Broth 2216 media was added to 10 L of seawater to a final concentration of 15 mg L^− 1^. The enriched seawater was incubated in the dark overnight at ambient temperature and gentle shaking. Aliquots of the enriched seawater were then sampled, and bacteria were recovered by differential centrifugation (4 °C, 4000 g for 20 min), then washed twice, and re-suspended in sterile, ice-cold CMFASW.

### Sponge RNA extraction, sequencing, and *de novo* transcriptome assembly

Total RNA from 30 samples was extracted for each species following the methods in Pita et al. [42], but only 29 and 22 samples of *A. aerophoba* and *D. avara*, respectively, passed the quality checks (i.e., RIN > 8 in Experion, Bio-Rad, USA) (Table 1). In short, 500 ng of total RNA were used for library construction with the TruSeq stranded mRNA library prep kit (Illumina, Inc., USA), including a poly-A enrichment step. Paired-end sequencing (150 bp) was performed on a NovaSeq S2 system (Illumina, Inc., USA) at the Competence Centre for Genomic Analysis (CCGA; Kiel, Germany). Raw paired-end reads were trimmed and filtered to remove adapters and low-quality reads in Trimmomatic-v0.39 [57]. Prokaryotic and microbial eukaryotic reads were filtered in the classifier kaiju-v1.6.2 [58]. All samples were used to construct a *de novo* assembly for each sponge species in Trinity-v2.10.0 [59]. Quality check and completeness of the assemblies were assessed by statistics performed in TransRate-v1.0.2 [60], and by comparing the assemblies against the metazoan-reference data in BUSCO-v3 (metazoan_odb9) [61].

### Annotation, gene quantification, and differential gene expression analysis

Functional transcriptome annotation was performed following Trinotate-v3.2.0 [59]. Contigs with Blastx or Blastp matches to Bacteria, Archaea, or Virus, as well as those annotated as ribosomal RNA were removed from the *de novo* assembly. Gene (i.e., trinity components) abundance was estimated based on RSEM bowtie2 quantification-v1.3.3 [62, 63]. Differential gene expression analysis was performed separately for each time point (i.e., 1 h, 3 h, and 5 h) in edgeR [64] as implemented in Trinity-v2.10.0 [59] with default parameters. Differentially expressed genes (DEGs) in pairwise- treatment comparisons were defined by False Discovery Rate-corrected (FDR) p-value < 0.005 and log2|change| ≥ 2 (i.e., four-fold change) as in [42, 65]. We defined DEGs as up- or down-regulated in the symbiont treatment when compared to the expression levels in the seawater bacteria treatment. In that sense, we considered the seawater bacteria treatment as our reference level (from now on termed “control”).

## Results

We characterized the transcriptomic response of the Mediterranean sponges *A. aerophoba* and *D. avara* to symbiont consortia extracted from *A. aerophoba* tissue compared to microbial consortia enriched from seawater. We followed upon the initial work by Wehrl et al. [32], who observed lower uptake rates of symbionts than seawater bacteria in *A. aerophoba* and no differential take up rates between bacteria types in *D. avara.*

### Reference transcriptome assembly

We sequenced 29 samples of *A. aerophoba* and 22 samples of *D. avara* corresponding to 3–5 biological replicates per treatment within 1 h, 3 h, and 5 h (Table 1). The number of paired-end Illumina reads generated in this study is summarized in Supplementary Table S1. BUSCO assessments revealed that the *de novo* reference transcriptomic assembly of *A. aerophoba* generated in this study contained 71.4% of the 902 BUSCO Metazoan core genes, with 76.6% of the genes found as complete. The reference transcriptome assembly for *D. avara* consisted of 78.2% of the BUSCO Metazoan core genes, with 82.9% of these genes found as complete. These reference transcriptomes are more complete than the reference reported by Pita et al. [42] for the same species, in which 69% and 70% of the genes were detected as core Metazoan genes in *A. aerophoba* and *D. avara*, respectively. Other transcriptomes [43, 66, 67] in different sponge species showed completeness regarding conserved BUSCO genes ranging from 87 to 99%. All statistics of the reference assemblies generated in this study are summarized in Supplementary Table S2. Overall, 68.89 ± 0.21% and 84.37 ± 17% (average ± standard error) of the reads in each sample aligned to the *de novo*-assembled reference transcriptome of *A. aerophoba* and *D. avara*, respectively (Table S3).

**Table 1:** Biological replicates per condition and time point. A total of 10 individuals were collected per species. Each individual was divided into three species and assigned to each sampling time point

Species	Treatment	Time	Replicates
*A. aerophoba*	Seawater microbial consortia	1 h	4
		3 h	5
		5 h	5
	*A.aerophoba* symbiont consortia	1 h	5
		3 h	5
		5 h	5
*D. avara*	Seawater microbial consortia	1 h	4
		3 h	4
		5 h	3
	*A.aerophoba* symbiont consortia	1 h	4
		3 h	3
		5 h	3

### Transcriptomic changes in response to symbiont consortia

Differentially expressed genes (DEGs) were defined by edgeR, using a threshold of log2|FC| ≥2 (i.e., 4-fold change) and FDR p-value < 0.005, as in previous studies [42, 65]. The DEGs were classified as up-regulated and down-regulated in the symbiont treatment when compared to the expression levels in the seawater microbial treatment (i.e., the latter treatment served as reference level). The results from the differential expression analysis in edgeR and the full Trinotate annotation reports for the DEGs can be found in Tables S4 to S8.

### *D. avara* transcriptomic response to symbiont consortia involves immune- and ubiquitin-related genes

**Fig. 1:**
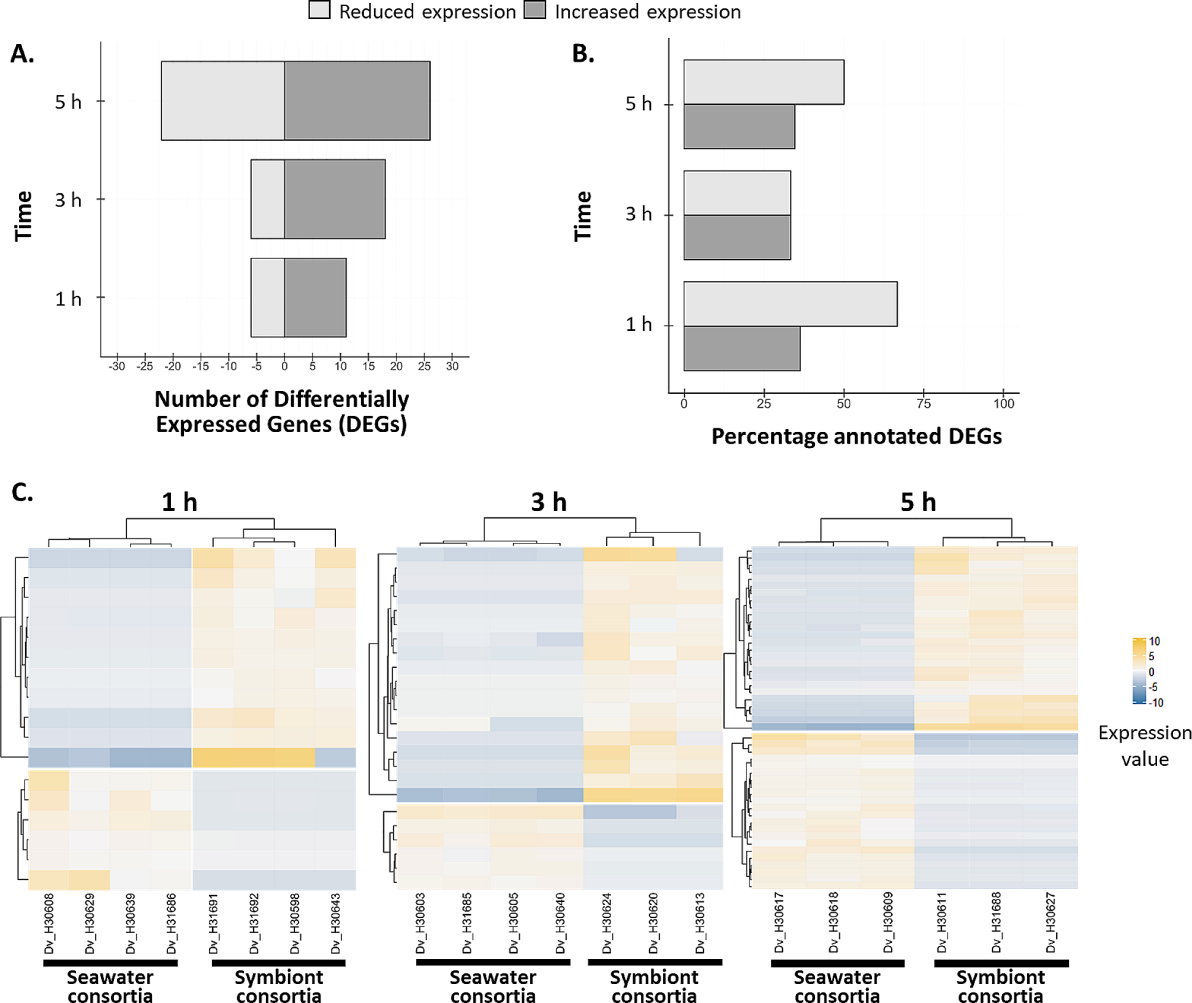
Differential gene expression of *D. avara* individuals treated with *A. aerophoba*-symbiont consortia relative to the seawater microbial consortia treatment. **(A)** Number of differentially expressed genes (DEGs). Genes with increased (dark gray) and reduced (light gray) expression upon symbiont encounter compared to seawater microbial consortia have positive and negative values, respectively. **(B)** Percentage of DEGs with annotation for each microbial treatment and time point. **(C)** Heatmaps show the relative expression level per DEG (rows) for each sample (columns) at 1 h, 3 h, and 5 h after microbial treatment. Dendrograms show gene clusters with similar expression patterns. Expression values are log2-transformed median-centred TMM-normalized values (color gradient). Genes were defined as differentially expressed with edgeR, FDR p-value < 0.005 and log2|FC|≥2.

We detected a total of 89 DEGs between *D. avara* sponges exposed to seawater and *A. aerophoba*-symbiont consortia and most genes showed higher expression levels in the symbiont treatment than in the control with seawater microbes (Fig. 1). The highest proportion of DEGs was detected at 5 h (Fig. 1A). Blastp provided annotation for approx. 40% of the total DEGs (Fig. 1B) and Trinotate annotation (based on the SignalP database: https://services.healthtech.dtu.dk/services/SignalP)classified all of them as non-transmembrane signaling peptides (Table S5). Expression profiles of *D. avara* individuals treated with each type of microbial consortia were consistent and biological replicates clustered together at all time points (Fig. 1C).

**Fig. 2:**
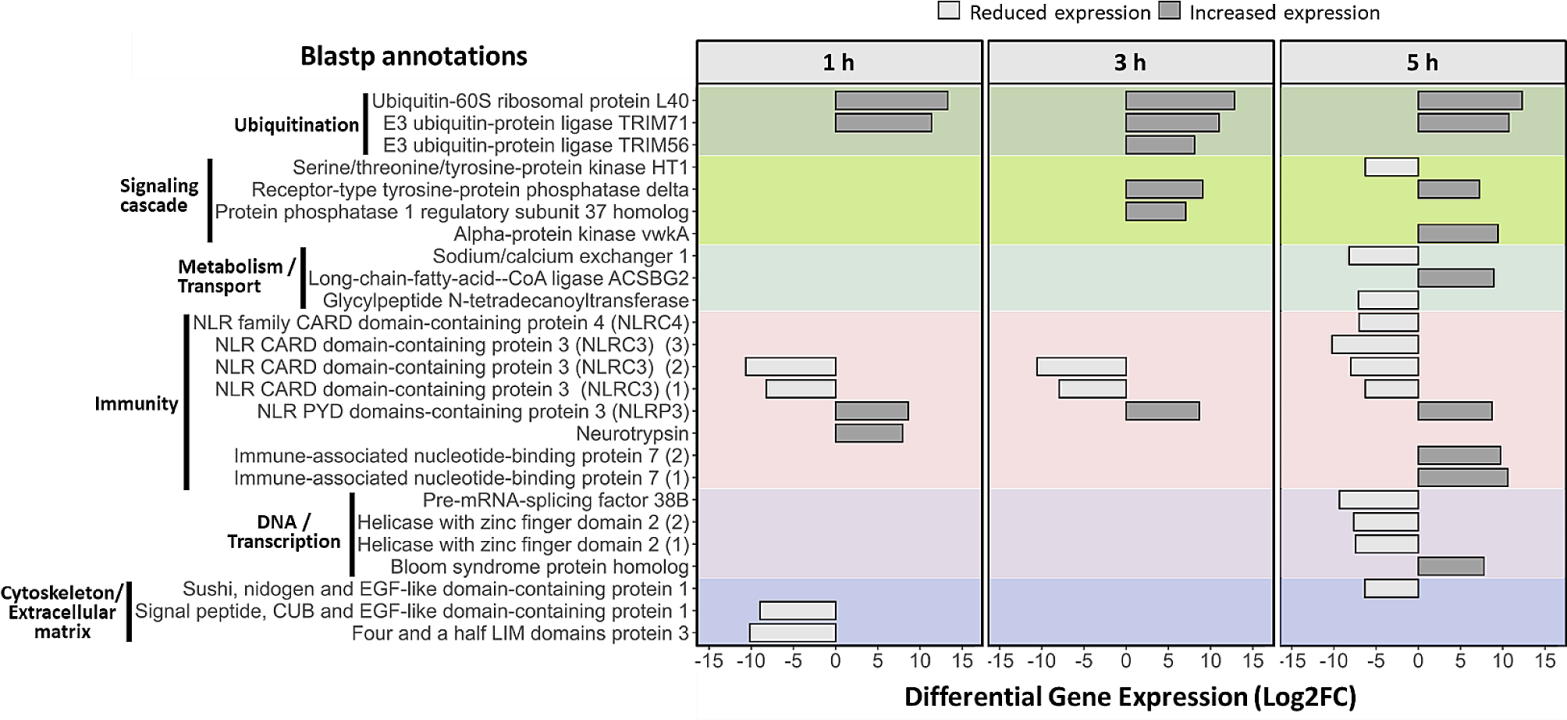
Functions and expression levels of differentially expressed genes in *D. avara* at 1 h, 3 h, and 5 h after *A. aerophoba*-symbiont consortia treatment relative to the seawater microbial consortia treatment. Genes with increased (dark gray) and reduced (light gray) expression upon symbiont encounter compared to seawater microbial consortia have positive and negative Log2FC values, respectively. Genes were defined as differentially expressed with edgeR, FDR p-value < 0.005 and log2|FC|≥2. Only genes with Blast annotations are included. Numbers in brackets indicate different genes with the same annotation

Based on Pfam and blast annotations, we identified five differentially expressed genes encoding for NOD-like receptors (NLRs) (Fig. 2, within “immunity” category). Four out of five differentially expressed NLRs showed lower expression levels upon exposure to *A. aerophoba*-symbionts than seawater microbes in *D. avara*. Three of these (*TRINITY_DN18609_c0_g1, TRINITY_DN65570_c0_g1, TRINITY_DN18609_c0_g2*) were expressed at all time points and corresponded to incomplete NLRs (only the LRR-domain was detected; PF13516), and annotated as NLRC3 based on Blastp, whereas the fourth gene (*TRINITY_DN6063_c1_g1*), found only at 5 h (Fig. 2), contained the characteristic NACHT domain of NLRs (PF05729) and a peptidase domain (PF00656), and was assigned to the NLRC4 family based on Blastp annotation (Table S5). In contrast, there was one NLR that showed elevated gene expression in sponges incubated with symbionts at all time points (*TRINITY_DN42758_c1_g2*); it contained a NACHT domain and was assigned to the NLRP3 family based on Blastp annotation (Fig. 2; and Table S5).

To confirm if these NLRs belonged to different subfamilies, we performed an additional blast search (at protein level, e-value < 1e − 5; Table S6) of the differentially expressed NLRs in *D. avara* against the freshwater sponge *Ephydatia muelleri*, for which a chromosome-level genome is available [68]. A phylogenetic analysis of these NLRs was not possible because our transcripts for these NLR-like genes were incomplete (i.e., lacking NACHT or LRR domains). The best hits of differentially expressed *D. avara* NLRs in *E. muelleri* support that the NLRs with increased expression in response to the symbiont treatment expression compared to the seawater microbial treatment belong to a different NLR subfamily than the one showing reduced gene expression (Table S6).

In addition to NLRs, we detected other DEGs potentially involved in innate immunity and ubiquitination that showed higher expression levels upon encounter to *A. aerophoba*-symbionts than to seawater microbes (Fig. 2). Among the immune genes, we detected an SRCR-containing gene associated to neurotrypsin (*TRINITY_DN137847_c3_g1*; PF00530), and two genes related to an immune-associated GTP-binding protein (PF04548) (*TRINITY_DN1745_c0_g1* and *TRINITY_DN5077_c0_g1*) (Fig. 2; and Table S5). The regulation of ubiquitination was evident by the differential expression of three genes: one ubiquitin-60 S ribosomal protein L40-like (*TRINITY_DN322946_c0_g1*) and two E3 ubiquitin ligases (*TRINITY_DN739_c0_g4* and *TRINITY_DN37530_c0_g1*, Fig. 2; and Table S5). The *A. aerophoba*-symbiont consortium also increased the expression of genes annotated as protein phosphatases (*TRINITY_DN4437_c1_g1* and *TRINITY_DN33881_c0_g1*) with fibronectin (PF00041) or LRR (PF13516) domains, an alpha-protein kinase (*TRINITY_DN61539_c1_g1*), a CoA ligase (*TRINITY_DN2791_c1_g1*), and a DEAD box-containing protein (*TRINITY_DN9624_c0_g1*; PF00270) (Fig. 2; and Table S5).

*D. avara* response to the symbiont treatment further involved lower expression levels of genes related to cell surface and cytoskeleton organization compared to the seawater microbial consortia treatment, including a calmodulin-ubiquitin and epidermal growth factor-like containing gene (*TRINITY_DN5241_c0_g1*), and a LIM domain-containing gene (*TRINITY_DN182729_c0_g1*) (Fig. 2 and Table S5). At 5 h, genes related to functions such as DNA regulation and transcription, metabolism and transport, and signaling cascades also showed reduced gene expression levels in the symbiont treatment. For example, two genes (*TRINITY_DN9504_c0_g1* and *TRINITY_DN41910_c0_g1*) for helicases with a zinc finger domain (HELZ2) belonging to the superfamily of P-loop NTPases (PF13087 and PF04851) which are predicted to be nuclear co-activators of the peroxisome proliferator-activated receptors (Fig. 2 and Table S5) were identified. We also detected two genes (*TRINITY_DN9504_c0_g1* and *TRINITY_DN41910_c0_g1*) involved in the molecular function of calcium and calmodulin binding. One of these genes contained a nidogen-like domain (PF06119), which is predicted to enable Notch binding activity and to be involved in cell-matrix adhesion, whereas the other gene with a Calx-beta motif (PF03160) regulates the transport of calcium and sodium across the cell membrane. In addition, a serine/threonine tyrosine-protein kinase (*TRINITY_DN63_c1_g1*), a glycylpeptide N-tetradecanoyltransferase (*TRINITY_DN16852_c2_g2*) involve in lipid modification, and an mRNA-splicing factor (*TRINITY_DN150336_c1_g1*) showed as well lower gene expression levels 5 h after the *A. aerophoba*-symbiont treatment than in the seawater microbial consortia treatment (Fig. 2 and Table S5).

### *A. aerophoba* differential response to symbiont consortia involves signaling genes, ubiquitination-related genes and kinases

**Fig. 3:**
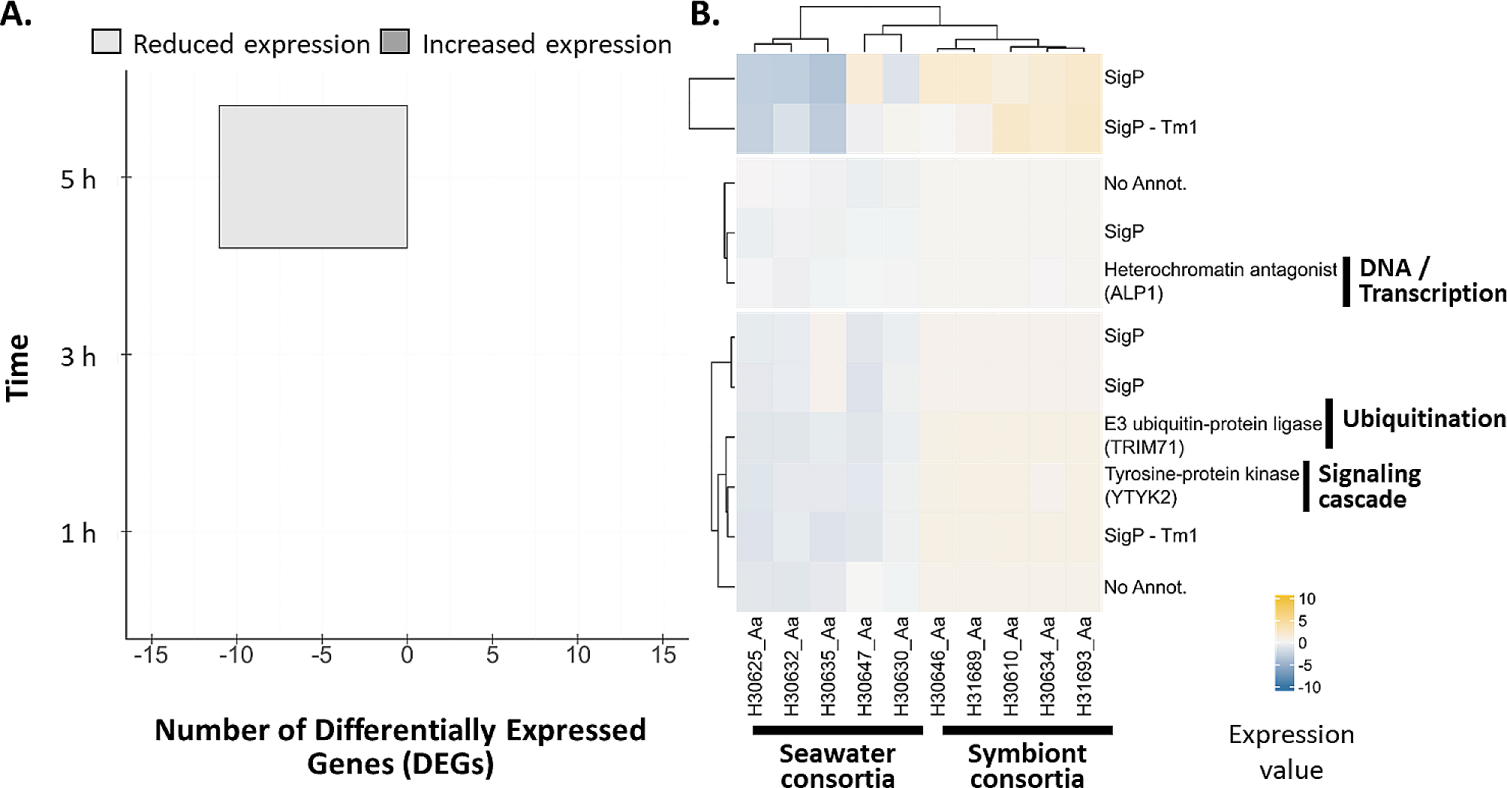
Differential gene expression of *A. aerophoba* individuals treated with the native sponge microbial consortia relative to the seawater microbial consortia treatment. **(A)** Number of differentially expressed genes (DEGs). Genes with increased (dark gray) and reduced (light gray) expression upon symbiont encounter compared to seawater microbial consortia have positive and negative values, respectively. **(B)** Heatmap shows the relative expression level per DEG (rows) for each sample (columns) at 5 h after microbial treatment. Dendrograms show gene clusters with similar expression patterns. Expression values are log2-transformed median-centred TMM-normalized values (color gradient). Functions of DEGs are included only for genes with Blast annotations (right bold legend). Genes were defined as differentially expressed with edgeR, FDR p-value < 0.005 and log2|FC|≥2.

**Fig. 4:**
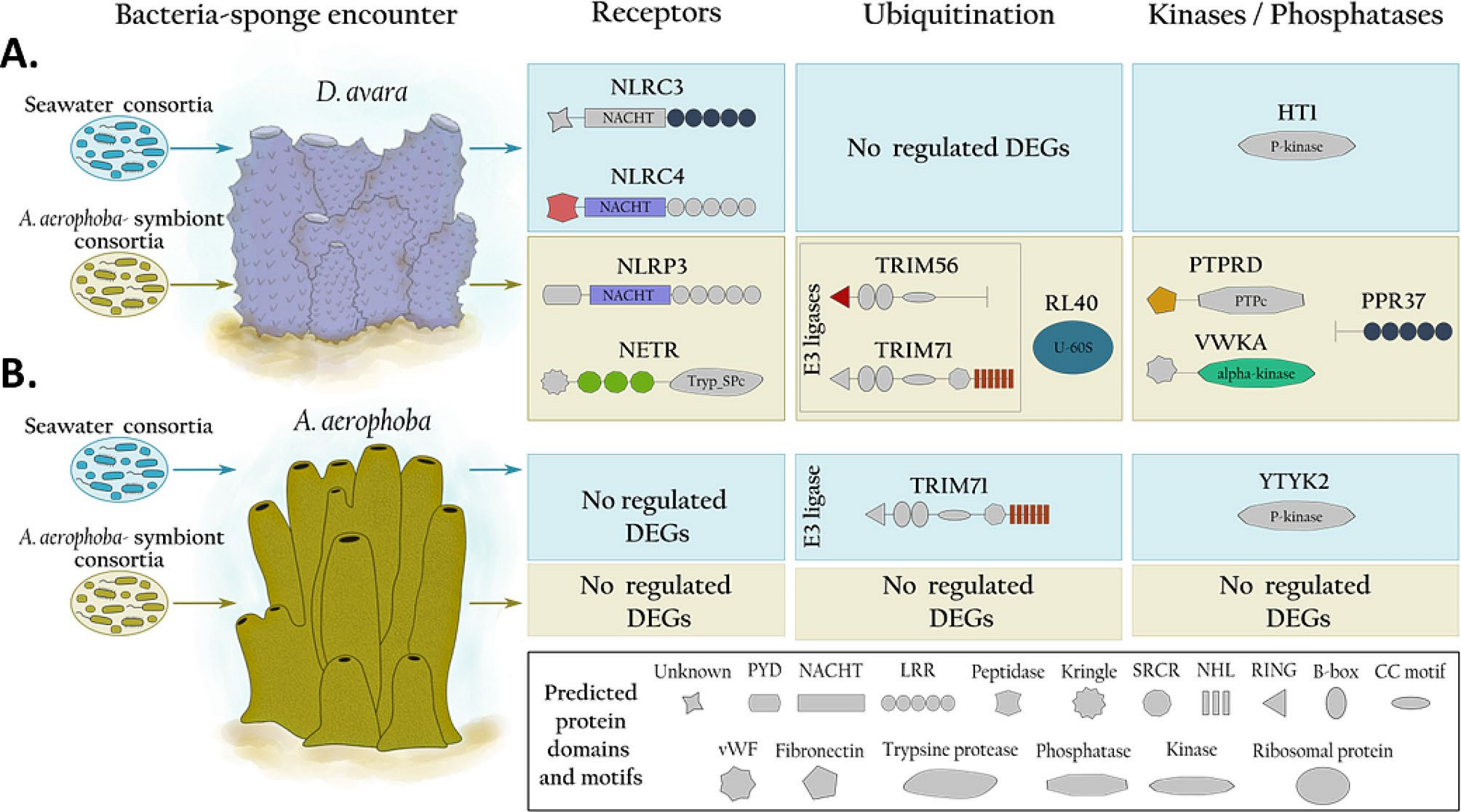
Overview of the transcriptomic response in **(A)***D. avara* and **(B)***A. aerophoba* upon encounter with *A. aerophoba-*symbiont consortia relative to the seawater microbial consortia treatment. DEGs with increased (yellow shade) and reduced expression (blue shade) in the symbiont treatment and annotated as receptors or related to ubiquitination and signaling are shown along with the UniProt ID of best blastp hits. Colored domains had a Pfam annotation, whereas gray-shaded ones were not detected, and the potential structure of each gene was drawn based on smart.embl.de

Differential gene expression was observed between *A. aerophoba* sponges incubated with symbiont consortia in comparison with the control treatment with seawater microbes but only after 5 h, (log2|FC| ≥2 (i.e., 4-fold change) and FDR p-value < 0.005), (Fig. 3A). Expression profiles consistently showed reduced levels in the symbiont treatment than in the incubations with seawater microbes(Fig. 3B). Within the total 11 DEGs detected, 9 genes were encoding hypothetical proteins containing a predicted signal peptide, as reported by SignalP database (https://services.healthtech.dtu.dk/services/SignalP) (and two contained a transmembrane domain Table S8). We identified three genes with additional blast annotation. A leucine-rich repeat receptor like protein kinase (*TRINITY_DN146410_c5_g2*) with similarity to a *Dictyostelium discoideum* gene (YTYK2; DDB_G0283397), an ubiquitin ligase (*TRINITY_DN163315_c3_g2*; LIN41), and a transposase-derived protein antagonist of heterochromatin (*TRINITY_DN169091_c1_g1*; ALP1) (Fig. 4B). When relaxing the significance threshold (log2|FC| ≥1 (2-fold change) and FDR p-value < 0.05), the number of DEGs increased. However, the pattern of reduced expression levels of DEGs in the symbiont consortia than seawater microbial treatment remained consistent (Fig. S2).

## Discussion

In this study, we characterized the transcriptomic responses of the Mediterranean sponges *A. aerophoba* and *D. avara* upon incubation with *A. aerophoba*-symbiont consortia compared to seawater microbial consortia. Previous studies showed that sponges take up seawater bacteria at higher rates than symbiotic bacteria [32–34] and that *A. aerophoba*-symbionts were incorporated into *D. avara* mesohyl but not into *A. aerophoba* mesohyl [33]. Accordingly, we report here that their transcriptomic response to symbiont compared to control treatment with seawater microbial consortia also differed. Notably, *D. avara* responded to the symbiont treatment mainly via the down-regulation of NLR receptors, whereas no PRRs were differentially regulated in *A. aerophoba*. This is among the first studies reporting differential regulation of various NLR families upon microbial exposure in sponges. While differentially expressed genes in *D. avara* showed higher levels of gene expression upon symbiont consortia encounter than when incubated with seawater microbial consortia, little differential expression was observed in *A. aerophoba*.

### Moderate transcriptional response of sponges to microbial exposure

The exposure of *A. aerophoba* and *D. avara* to *A. aerophoba-*symbionts in comparison with seawater microbes showed differential gene expression of few genes (i.e., < 70 genes; Figs. 1A and 4A), even when significance threshold was relaxed (Fig. S2). We detected a relatively lower transcriptional response (i.e., number of DEGs) than in previous studies, particularly for *A. aerophoba.* A previous experiment assessing the response of both sponge species studied here to commercial microbial elicitors (i.e., lipopolysaccharide and peptidoglycan), compared to a sham injection with filtered artificial seawater, detected > 400 DEGs and approx. 49 DEGs in *A. aerophoba* and *D. avara*, respectively [42]. In another study, the transcriptional response of *A. aerophoba* to wounding included thousands of DEGs [65]. Besides potential differences due to experimental design or analysis, we propose that, to some extent, the magnitude of the host response is scalable depending upon the treatment, ranging from low (exposure to natural bacterial consortia) to high (mechanical damage). The constant interactions of sponges with their microbiome and seawater bacteria, including potential pathogens, may favor a “fine-tuning” of sponge baseline gene expression over induced activation of immune components in response to microbes. Thus, major transcriptomic shifts will only occur in cases of homeostasis disruption as observed in the response of *A. aerophoba* to wounding [65]. This strategy challenges traditional views on microbial-induced immunity in terrestrial animals, but it may indeed be widespread among marine invertebrates [43, 69].

### Sponges respond to symbiont microbial consortia differently

In *D. avara* individuals incubated with *A. aerophoba*-symbiont consortia we observed a differential expression of immune receptors such as NLRs (Figs. 2 and 3A), whereas no PRRs were differentially expressed in *A. aerophoba* (Figs. 3B and 4B). The direction of gene expression changes was also opposite in the two species: in *D. avara*, symbiont treatment mainly resulted in upregulation of genes when compared with incubations with seawater bacteria (Fig. 1C), whereas the few DEGs in *A. aerophoba* were mainly down-regulated in symbiont treatment in relation to seawater bacteria treatment (Fig. 4B). We speculate that *A. aerophoba* may be recognizing its own symbionts as “native/known” while *D. avara* may be sensing *A. aerophoba*-symbionts as “non-self/unknown” “foreign” microbial consortia. Ideally, we would have included an additional treatment consisting of *D. avara*-symbiont consortia exposure to clarify these hypotheses, but we were not able to extract symbionts from *D. avara* in sufficient quantity for our incubations, due to its LMA status. Moreover, we cannot exclude the possibility that *A. aerophoba* sponge cells were remaining in the microbial symbiont fraction and, thus, affected the transcriptional responses. However, we would expect that those sponge cells will activate a transcriptional response in both *D. avara* and *A. aerophoba*, if any, because studies on sponge self- and non-self-transplants suggest active rejection of cells from other sponges, even if derived from other individuals of the same species [70–72]. An alternative explanation to the differential response between the sponge species may be related to the host intrinsic characteristics such as species-specific traits or to the HMA-LMA status. More sponge species representative of the HMA-LMA categories will however be needed to discern species-specific responses from different immune strategies related with the HMA-LMA status.

### *D. avara* and *A. aerophoba* response to symbionts involves different sets of genes

The differential transcriptomic response of *D. avara* individuals to *A. aerophoba*-symbionts compared to seawater microbial consortia involved several immune genes including one SRCR-containing receptor and two GTP-binding proteins. The genes encoding for the immune-associated GTP-binding protein (IAN) in *D. avara* are part of the GIMAP family. IAN genes were not detected in any other early divergent metazoans including the genome of the sponge *Amphimedon queenslandica*, but are broadly and patchy distributed among eukaryotes, and orthologs have been reported in plants, corals, and molluscs as means of microbial defense [73–76]. GIMAP family is conserved among vertebrates, where it is implicated in the development and maintenance of immune cells (e.g., lymphocytes [77]). SRCRs are involved in the recognition of a broad range of ligands and are highly diversified in invertebrates [18, 21, 78]. These receptors are also expanded in sponges [42, 43, 79] and potentially play a role in sponge symbiosis. For instance, a SRCR-domain containing gene was up-regulated in symbiotic individuals of the sponge *Petrosia ficiformis* compared to aposymbiotic individuals (i.e., photosymbiont-free), suggesting the involvement of this immune receptor in sponge symbiosis [80]. Moreover, in juveniles of *A. queenslandica*, different SRCRs were upregulated in response to native and foreign bacteria [41]. Altogether, SRCRs arise as putative mediators of sponge-microbe interactions in different sponge species and the GIMAP family may as well deserve more attention in future studies.

In comparison to *D. avara*, the lower differential transcriptomic response of *A. aerophoba* to its own symbionts was limited to reduced expression of a kinase-like receptor, an E3 ligase and an antagonist of heterochromatin compared to the seawater microbial consortia treatment (Figs. 3B and 4B). The antagonist of like-heterochromatin protein (ALP1) in plants acts as a transposase mediating various cellular pathways and capable of silencing gene expression involving E3 ubiquitin ligases [81, 82]. The role of this transposase in inhibiting transcriptional responses is proposed to have evolved as a means for evading surveillance by the hosts [83]. In fact, pathogens cause a variety of transcriptional changes (e.g., alteration of chromatin structure, proteolytic degradation, deactivation of transcription factors, etc.) to exploit a wide range of pathways which enhances their survival within the host [84, 85]. We therefore hypothesize that the late (i.e., at 5 h) reduced gene expression of both ALP1 and E3 ubiquitin ligase in symbiont compared to seawater microbial exposure (Fig. 4B) could indicate active host gene silencing by symbionts, to prevent becoming target material for degradation. Functional studies are imperative to validate the processes in which the detected DEGs are involved, yet this remains a challenge in sponges as models for genetic manipulation are currently limited to explants or cells of two sponge species [86, 87].

### *D. avara* responds to microbial consortia via NLRs

*D. avara* specimens responded to *A. aerophoba-*symbiont consortia via differential expression of NLRs. The first experimental evidence of enhanced NLR expression in sponges was reported in *D. avara* as a response to commercial microbial elicitors [42]. Our results build on these observations and suggest synergies between different NLRs for specific microbial recognition. In juveniles of *A. queenslandica*, the response to native (symbionts) and foreign (non-symbiont) bacterial consortia involved the upregulation of an NLR compared to non-treated control specimens [41]. In our study, NLRs with lower expression levels in sponges incubated with *A. aerophoba*-symbionts, compared to sponges treated with seawater microbial consortia, were similar to the NLRC3 and NLRC4 families (based on Blastp results; Figs. 2 and 3A), whereas the NLR with higher expression levels in symbiont treatment showed similarity to the NLRP3 family (Figs. 2 and 3A).

Differential gene expression of NLRs in *D. avara* was accompanied by differential expression of ubiquitination, kinases and phosphatases (Figs. 2 and 3A). We speculate that the different types of NLRs (i.e., NLRC3, NLRC4 and NLRP3) activate different downstream signaling pathways in *D. avara* whose ultimate goal is to regulate microbial recognition by the sponge. In humans and mice these NLR families regulate inflammatory pathways [26, 88–91]. Inflammation requires various post-translational modifications comprising ubiquitin ligases, kinases and phosphatases [92–94], and the ubiquitin system, which is crucial in many biological process, is proposed as an essential innate immunity regulator and as a modulator of host-microbe interactions [95, 96].

The regulation of NLRs we observed in the LMA sponge *D. avara*, compared to the non-regulation of these receptors in the HMA sponge *A. aerophoba*, may further support the hypothesis that these receptors potentially regulate the interaction between LMA sponges and microbes (e.g., [37, 42, 43]). Importantly, LMA sponges are known to contain an expanded and diverse set of NLRs compared to HMAs (e.g., [37, 43, 79, 97]). Our results further show experimentally the potential role of differential transcription of NLRs in microbial discrimination by LMA sponges. Future studies should focus on identifying the ligand of these different NLRs to finally provide functional evidence of the role of sponge NLRs in immune specificity.

## Conclusion

The molecular mechanisms employed in early divergent metazoans for microbial discrimination are still only poorly understood. In the present study, we characterized the transcriptomic response of two sponge species upon exposure to sponge-derived symbionts, in comparison to incubations with seawater microbial consortia, by RNA-Seq differential gene expression analysis. The relatively low number of DEGs detected in both *D. avara* (LMA) and *A. aerophoba* (HMA) specimens incubated with microbial consortia may suggest that sponges respond to microbes by favoring fine regulation over induced gene activation due to their constant interactions with their microbiome and seawater bacteria. The sponge *D. avara* regulated the expression of different families of NLR-like genes upon exposure to *A. aerophoba*-symbionts, while *A. aerophoba* showed little differential gene expression and no participation of PRRs upon exposure to its symbionts. We hypothesize that the different NLRs under regulation act in synergy in *D. avara* to recognize between different microbial cues. Furthermore, we propose that the differential response to *A. aerophoba*-symbiont treatment between sponge species could be the result of [1] “foreign” *vs. “*native” recognition of the microbial consortia by *D. avara* and *A. aerophoba*, respectively and/or [2] the repertoire of immune genes harbored by the host and the degree to which these are induced. The latter explanation may be related to species-specific traits, as well as to the HMA-LMA status of the sponges. To unveil more potential sponge molecular adaptations to microbial encounters it is crucial to investigate different sponge species along the LMA-HMA spectrum, under experimental setups that resemble as much as possible natural conditions, and to test different microbial structures that may induce or silence the transcriptomic response of the host. Finally, conducting comparative studies between the relevant genes mediating microbial discrimination in sponges and other early divergent invertebrates would further expand our understanding on the role of PRRs on microbial recognition and place sponges with their unique life-styles in an evolutionary context.

### Electronic supplementary material

Below is the link to the electronic supplementary material.

Supplementary Material 1

Supplementary Material 2

Supplementary Material 3

## Data Availability

The raw reads, metadata, transcriptome assembly and full annotation for this study have been deposited in the European Nucleotide Archive (ENA) at EMBL-EBI under the accession number PRJEB61959 (ERP147040).
