# Supplementary material for: Transcriptomic responses of Mediterranean sponges upon encounter with symbiont microbial consortia
Source: BMC Genomics. 2024 Jul 7;25:674. doi: 10.1186/s12864-024-10548-z (PMC11229196; doi:10.1186/s12864-024-10548-z)
Supplement: Supplementary file 2 — Supplementary Material 2 [file 12864_2024_10548_MOESM2_ESM.docx]

**Supplementary information**

**Text S1.** Characterization of microbial consortium treatments by flow cytometry

The concentration of the microbial consortia stocks obtained by enrichment was estimated via flow cytometry and adjusted to reach 10^5-6^ bacteria mL^-1^ final concentration in each experimental aquarium. In addition, water samples (2 mL) from each aquaria were collected right before the experiment (T-1h) and right after (T0h) adding the microbial consortium. Samples for flow cytometry were fixed in paraformaldehyde + glutaraldehyde (1% + 0.05% final, respectively) and stored at -80ºC until analysis. Microbial cell concentration was quantified by flow cytometry (FACSCalibur, Becton-Dickinson, 488 nm excitation blue laser) following the method of Gasol and Morán (1999). In short, DNA in microbial cells was stained with Syto13, and detected based on cell-side scatter, forward scatter, and green fluorescence of the stained DNA. Plastic beads were used as reference for plotting. Bacterial cell concentrations were calculated based on number of events and calibrated flow rate.

Although the aquaria were kept overnight in 1 µm-filtered seawater and an additional 0.1 µm-filter was applied for 3 h before the experiments, the water in the aquaria was not sterile, some bacterial cells remained (Fig. S1 A and C). We could still detect the addition of the treatment, particularly in the cell population of higher DNA content, in both seawater and *A. aerophoba* symbiont treatments (Fig. S1 B and D, R6 gate). We could detect and increment of one order of magnitude in the bacterial concentrations in the water before and after the addition of microbial consortia, to a final concentration ~10^6^ cells/mL.


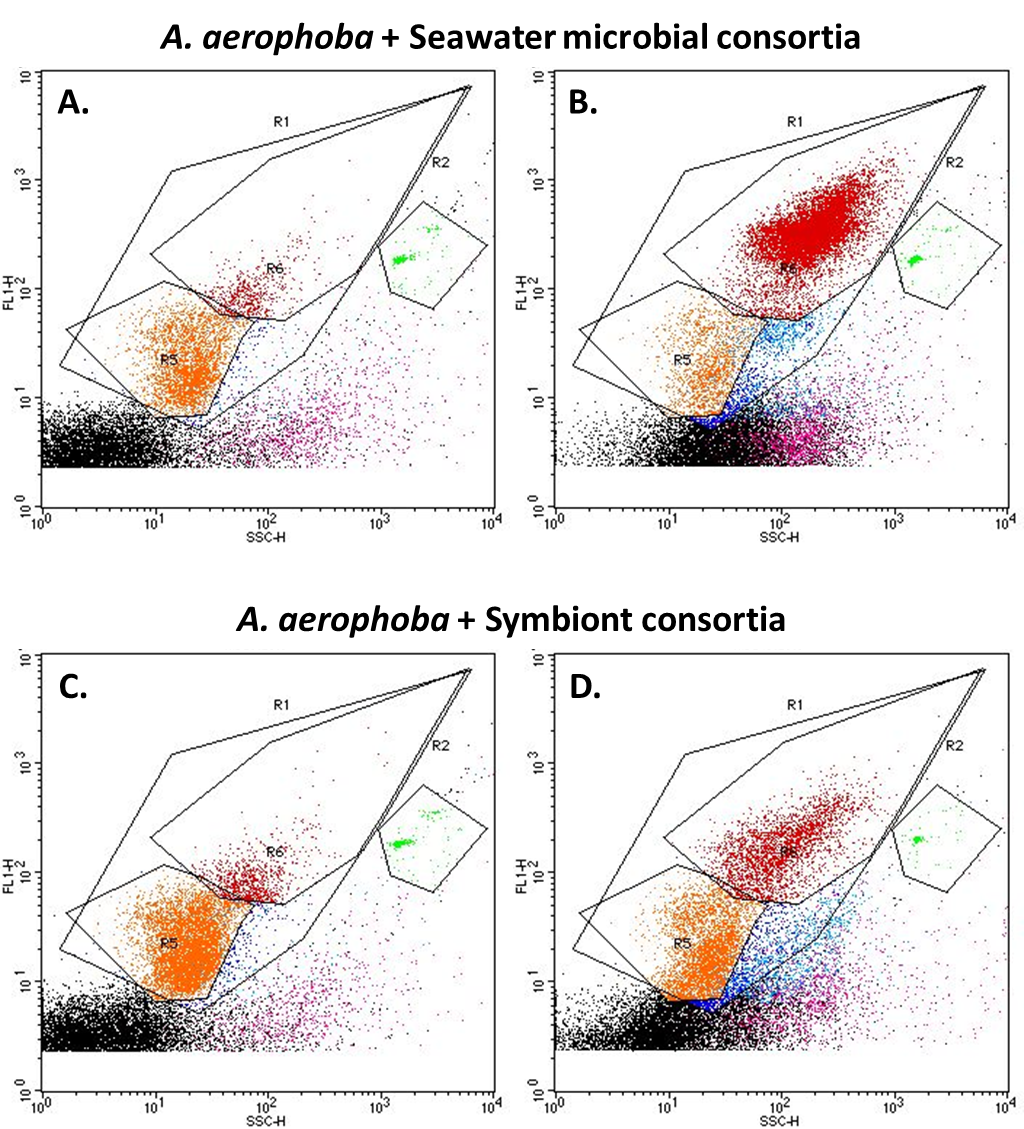


**Fig. S1.** Representative cytograms of seawater consortia **(A-B)** and *A. aerophoba*-symbiont **(C-D)** consortium used for the experiments. The microbial stock concentration was estimated before (T-1) **(A-C)** and after (T0) **(B-D)** adding the bacteria to the incubation tank. R1: all bacteria; R5 and R6: low and high DNA bacteria, respectively; R2: quantification beads. Water samples (2 mL) from all aquaria were collected before the experiment (time point -1h) and every hour during the course of the experiments (time points 0, 1, 2, 3, 4, 5 h), and fixed in paraformaldehyde + glutaraldehyde (1% + 0.05% final, respectively). Microbial cell concentration in the water by was quantified by flow cytometry (FACSCalibur, Becton-Dickinson, 488 nm excitation blue laser) following the method of Gasol and Morán (1999), to assess the sponge filtration activity. The bacterial cells were stained with Syto13, and detected based on cell-side scatter, forward scatter, and green fluorescence of the stained DNA. For comparison with the sponges, control aquaria (i.e., without sponge) were also exposed to the microbial treatments and sampled at the same time points.

**Table S1.** Number of read pairs (million reads). “Raw” refers to the output from sequencing; “Clean” to surviving read pairs after trimming and filtering in trimmomatic-v0.38; and “Eukaryote” to pairs identified as non-prokaryotic and nonmicrobial eukaryote by kaiju-v1.6.2 (Menzel & Krogh, 2015).

| **Average per library**  **(± standard error)** | **Raw** | **Clean** | **Eukaryote** |
| --- | --- | --- | --- |
| *A. aerophoba* | 23.8 ± 1.8 | 22.1 ± 9.2 | 15.0 ± 6.3 |
| *D. avara* | 19.6 ± 1.2 | 18.1 ± 10.7 | 11.7 ± 0.7 |

**Table S2.** Statistics of the *de novo* transcriptomic assemblies. Transcripts refer to Trinity isoforms, genes refer to Trinity components. Mb: mega bases.

| **Statistics:** | ***A. aerophoba*** | ***D. avara*** |
| --- | --- | --- |
| No. Transcripts – Trinity isoforms | 900127 | 983239 |
| No. Genes – Trinity components | 466345 | 624596 |
| Transcripts with open reading frames, % | 60.59 | 52.88 |
| Average transcript length, nucleotides | 535.16 | 636.86 |
| N50 | 631 | 873 |
| Total assembled bases, Mb | 481.7 | 626.2 |
| **BUSCO report**  **(metazoan database; 978 genes)** | **C:71.4%**  **[D:44.1%, F:23.6%]** | **C:78.2%**  **[D:49.0%,F:17.1%]** |

**Table S3.** Alignment rates of the *de novo* transcriptomic assemblies based on RSEM bowtie2 (v1.3.3).

| ***Aplysina aerophoba*** | |  | ***Dysidea avara*** | |
| --- | --- | --- | --- | --- |
| **File name** | **Overall alignment rates (%)** |  | **File name** | **Overall alignment rates (%)** |
| rsem_SWSY_Aa_H30597.out | 68.78 |  | rsem_SWSY_Dv_H30598.out | 85.23 |
| rsem_SWSY_Aa_H30599.out | 67.92 |  | rsem_SWSY_Dv_H30603.out | 84.86 |
| rsem_SWSY_Aa_H30604.out | 68.18 |  | rsem_SWSY_Dv_H30605.out | 83.14 |
| rsem_SWSY_Aa_H30606.out | 69.41 |  | rsem_SWSY_Dv_H30608.out | 84.34 |
| rsem_SWSY_Aa_H30610.out | 70.51 |  | rsem_SWSY_Dv_H30609.out | 84.49 |
| rsem_SWSY_Aa_H30615.out | 70.21 |  | rsem_SWSY_Dv_H30611.out | 85.41 |
| rsem_SWSY_Aa_H30616.out | 68.47 |  | rsem_SWSY_Dv_H30613.out | 84.32 |
| rsem_SWSY_Aa_H30619.out | 67.18 |  | rsem_SWSY_Dv_H30617.out | 84.3 |
| rsem_SWSY_Aa_H30622.out | 67.64 |  | rsem_SWSY_Dv_H30618.out | 84.96 |
| rsem_SWSY_Aa_H30623.out | 68.72 |  | rsem_SWSY_Dv_H30620.out | 84.94 |
| rsem_SWSY_Aa_H30625.out | 69.73 |  | rsem_SWSY_Dv_H30624.out | 84.39 |
| rsem_SWSY_Aa_H30630.out | 68.48 |  | rsem_SWSY_Dv_H30627.out | 84.45 |
| rsem_SWSY_Aa_H30632.out | 68.15 |  | rsem_SWSY_Dv_H30629.out | 85.37 |
| rsem_SWSY_Aa_H30633.out | 70.4 |  | rsem_SWSY_Dv_H30639.out | 84.93 |
| rsem_SWSY_Aa_H30634.out | 69.5 |  | rsem_SWSY_Dv_H30640.out | 85.36 |
| rsem_SWSY_Aa_H30635.out | 71.04 |  | rsem_SWSY_Dv_H30643.out | 83.41 |
| rsem_SWSY_Aa_H30636.out | 69.01 |  | rsem_SWSY_Dv_H31685.out | 83.49 |
| rsem_SWSY_Aa_H30637.out | 70.07 |  | rsem_SWSY_Dv_H31686.out | 83.07 |
| rsem_SWSY_Aa_H30638.out | 69.12 |  | rsem_SWSY_Dv_H31688.out | 83.59 |
| rsem_SWSY_Aa_H30641.out | 68.94 |  | rsem_SWSY_Dv_H31691.out | 84.86 |
| rsem_SWSY_Aa_H30642.out | 67.98 |  | rsem_SWSY_Dv_H31692.out | 82.95 |
| rsem_SWSY_Aa_H30644.out | 70.41 |  |  |  |
| rsem_SWSY_Aa_H30645.out | 69.91 |  |  |  |
| rsem_SWSY_Aa_H30646.out | 69.21 |  |  |  |
| rsem_SWSY_Aa_H30647.out | 68.41 |  |  |  |
| rsem_SWSY_Aa_H31689.out | 68.14 |  |  |  |
| rsem_SWSY_Aa_H31690.out | 68.96 |  |  |  |
| rsem_SWSY_Aa_H31693.out | 67.04 |  |  |  |
| rsem_SWSY_Aa_H31694.out | 66.38 |  |  |  |
| **Average** | **68.89** |  |  | **84.37** |
| **stand error** | **0.21** |  |  | **0.17** |

**Table S4.** Differential Expression analysis for *D. avara* at as identified in edgeR (FDR p-value < 0.005 and log2|FC|≥2) at 1h, 3h and 5h (Excel file)

**Table S5.** Annotation of the differentially expressed genes for *D. avara* identified in edgeR (FDR p-value < 0.005 and log2|FC|≥2) at 1h, 3h and 5h (Excel file)

**Table S6.** Blastp results of *D. avara* differentially expressed NLRs against *Ephydatia muelleri* (e-value < 1e−5) (Excel file)

**Table S7**. Differential Expression analysis for *A. aerophoba* at as identified in edgeR (FDR p-value < 0.005 and log2|FC|≥2) at 5h (Excel file)

**Table S8.** Annotation of differentially expressed genes for *A. aerophoba* identified in edgeR (FDR p-value < 0.005 and log2|FC|≥2) at 5h (Excel file)


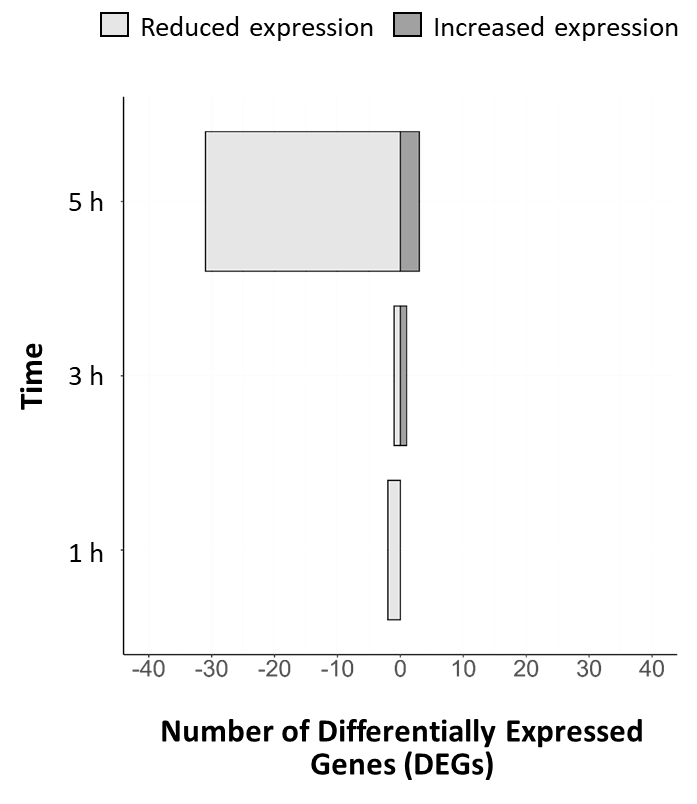


**Fig. S2.** Number of differentially expressed genes (DEGs) of *A. aerophoba* individuals treated with symbiont consortia compared to control treatment with seawater microbial consortia. Genes with increased (dark gray) and reduced (light gray) expression upon symbiont encounter compared to seawater microbial consortia have positive and negative values, respectively. Genes were defined as differentially expressed with edgeR, FDR p-value < 0.05 and log2|FC|≥1.
