# Supplementary figures and images for: Transcriptomic responses of Mediterranean sponges upon encounter with symbiont microbial consortia
Source: BMC Genomics. 2024 Jul 7;25:674. doi: 10.1186/s12864-024-10548-z (PMC11229196; doi:10.1186/s12864-024-10548-z)

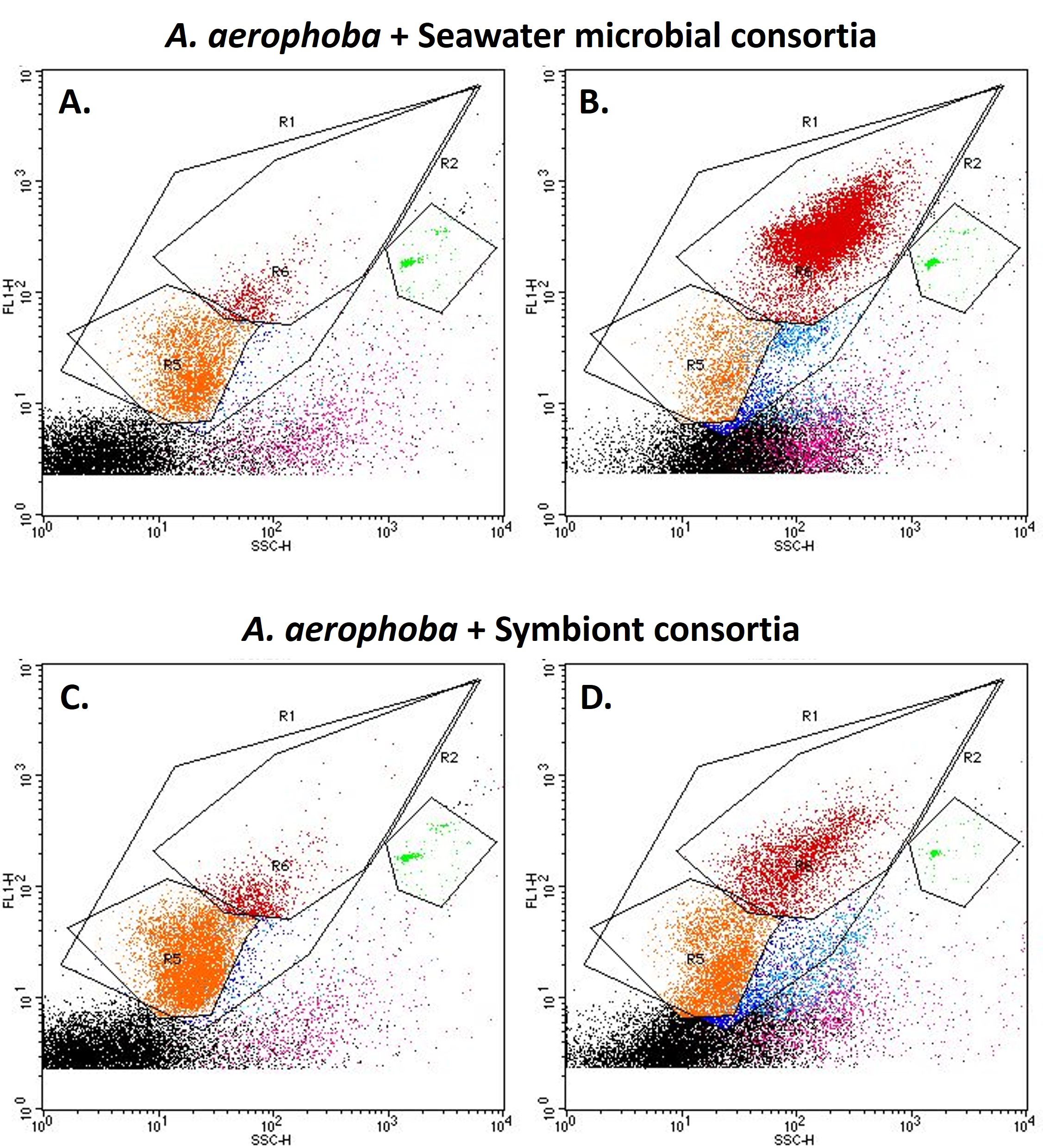

Supplement: Supplementary file 3 — Supplementary Material 3 [file 12864_2024_10548_MOESM3_ESM.jpg]
